# Finite Element Modeling on Shear Performance of Grouted Stud Connectors for Steel–Timber Composite Beams

**DOI:** 10.3390/ma15031196

**Published:** 2022-02-04

**Authors:** Henan Zhang, Zhibin Ling

**Affiliations:** School of Civil Engineering, Suzhou University of Science and Technology, Suzhou 215011, China; 1913031088@post.usts.edu.cn

**Keywords:** steel-timber, composite, shear connector, stud, grout

## Abstract

Steel–timber composite (STC) systems are considered as an environmentally friendly alternative to steel–concrete composite (SCC) structures due to its advantages including high strength-to-weight ratio, lower carbon footprint, and fully dry construction. Bolts and screws are the most commonly used connectors in STC system; however, they probably make great demands on the accuracy of construction because of the predrilling in both the timber slabs and steel girder fangles. To address this issue, the STC connections with grouted stud connectors (GSC) were proposed in this paper. In addition, stud connectors can also provide outstanding stiffness and load-bearing capacity. The mechanical characteristic of the GSC connections was exploratorily investigated by finite element (FE) modeling. The designed parameters for the FE models include stud diameter, stud strength, angle of outer layer of cross-laminated timber (CLT) panel, tapered groove configurations, and thickness of CLT panel. The numerical results indicated that the shear capacity and stiffness of the GSC connections were mainly influenced by stud diameter, stud strength, angle of outer layer of CLT panel, and the angle of the tapered grooves. Moreover, the FE simulated shear capacity of the GSC connections were compared with the results predicted by the available calculation formulas in design codes and literatures. Finally, the group effect of the GSC connections with multiple rows of studs was discussed based on the numerical results and parametric analyses. An effective row number of studs was proposed to characterize the group effect of the GSC connections.

## 1. Introduction

As a green and renewable building material, timber has the characteristics of light weight, high strength, and easy processing. Using steel–timber composite (STC) floors instead of conventional steel–concrete composite (SCC) floors can effectively reduce energy consumption and carbon emissions throughout the life cycle of the structures [1]. Compared with that of conventional SCC beams, the STC beams can decrease structural weight, seismic response, and cross-section of structural elements significantly [2]. The STC beams consist of an upper timber slab connected to the bottom steel beam with shear connectors. The shear connectors are mainly responsible for transmitting the shear force between the slab and the beam, and meanwhile, preventing vertical uplift between two materials. Several types of shear connectors were developed for STC floor system including dowel-type connectors (e.g., screws and bolts), dowels and adhesive composite connection, and bolted connectors embedded in grout pockets [3,4,5,6,7,8,9,10,11,12,13,14]. The existing research on STC shear connections mainly focuses on the conventional dowel-type connection. Hassanieh et al. [3,4] through push-out test studied the shear performance of steel-CLT (cross-laminated timber) and steel-LVL (laminated veneer lumber) connections with diverse types of connectors, including coach screw, dog screw, bolt, and screw and adhesive composite connection. Moreover, the behavior as well as composite efficiency of STC beams with the above-mentioned shear connections were analyzed by four-point bending tests [5,6]. Loss et al. [7,8,9] designed and tested different connections for steel–timber hybrid prefabricated systems, and it was reported that one of which was ideal solution for STC floor, ensuring a high load-bearing capacity and slip modulus, as well as ductility. Wang et al. [10] proposed the inclined self-tapping screws for STC joints to address problems of construction inconvenience and buried depth of screws. Ataei et al. [11] investigated the cyclic behavior of screw and bolt connectors reporting that the STC joints exhibited high ductility and energy dissipating capacity. Chiniforush et al. [12] studied the long-term performance of steel-CLT composite connections through push-out test and established a long-term rheological model that considered their predictions of the slip.

Although typical dowel-type connectors for STC beams (i.e., bolts and screws) demonstrated an ideal load–slip response, most of them need predrilling at both the steel beam flanges and timber slabs before connecting, which requires higher construction accuracy and is considerably inconvenient for installation. Accordingly, the grouted stud connectors (GSC) shear connections consisting of two main parts, the welded shear studs, and its surrounding grout were proposed for connecting the timber slab and the steel girder in STC beams. The studs are welded directly to the beam flange without predrilling, then grooving in CLT panel at the locations corresponding to the studs, and finally filling the groove with cement grout to form the GSC shear connections. Using GSC shear connections can effectively reduce the overhead work required to install conventional fasteners to construct STC floors and compensate for the lack of construction accuracy. Furthermore, Hassanieh et al. [6,13,14] proved the effectiveness of the STC joints with bolt connectors embedded in grout pockets (BCGP) through experimental and numerical studies. The results indicated that the BCGP connections presented better stiffness, bearing capacity, ductility, and composite efficiency compared to that of the conventional bolted or screwed STC connections, and the long-term behavior was also proved to be superior [12]. However, compared to that of shear studs, the BCGP connections might not be the most effective connecting methods for STC system because of the predrilling for the assembly of bolts; thus, the studs were expected to be an alternative, which were proved to be of outstanding load-bearing capacity and stiffness, as well as of convenient construction by abundant studies [15,16,17,18] and practical engineering. Consequently, the GSC shear connections for STC beams were exploratorily proposed in this study. FE modeling on the GSC shear connections was conducted using ABAQUS to investigate the shear performance of the connections; further, the influences of the stud diameter, stud strength, grain directions of timber, configurations, angles of grouting groove, and thickness of CLT panel on the load–slip response, peak load capacity, and stiffness were also studied. The results of this study can provide some references for the design of STC connections.

## 2. FE Modeling and Verification

### 2.1. Geometrical Parameters

**Figure 1:**
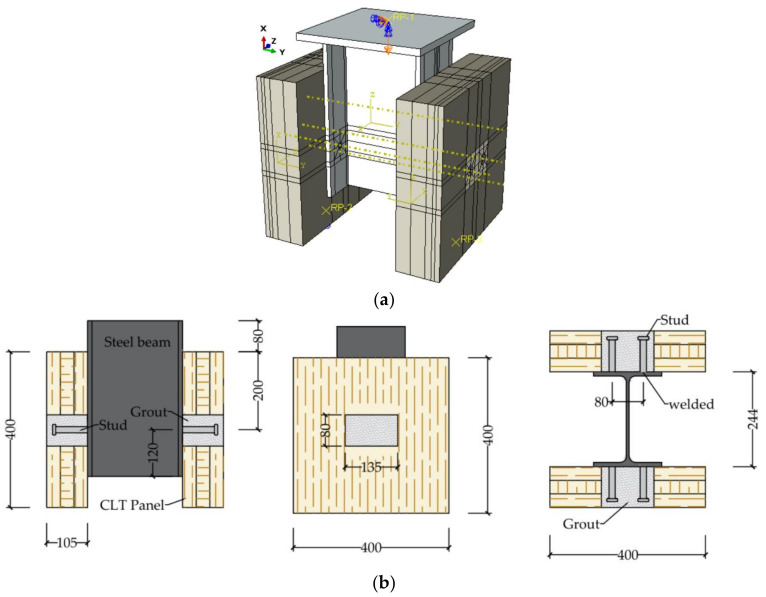
Outline of GSC shear connection, (**a**) solid model; (**b**) Geometry, cross-section and details. (Unit: mm).

The GSC shear connection specimens were designed to experience double shear push-out tests to evaluate the shear performance of the connections. FE models were established by ABAQUS [19] in this study. Figure 1a,b show the FE solid model and the geometric dimensions of the designed specimens, respectively.

### 2.2. Material Constitutive Laws and Properties

#### 2.2.1. Timber

The timber species used in this study is Spruce, which is assumed to be homogeneous and anisotropic, while isotropic at the two orientations perpendicular to the grain, indicating the same mechanical properties in radial and tangential directions [20]. Considering the orthogonality of the CLT panel composed of crossed lamellae layers, the mechanical properties and strength of timber were assigned to each layer separately. The mechanical properties of timber used in the FE model are referenced in the literature [14] since the same timber type is used. Table 1 reports the modulus of elasticity and Poisson’s ratio of the timber. Table 2 presents the compressive strength $f_{c}$ , tensile strength $f_{t}$ , shear strength $f_{s}$ , and fracture energy $G_{f}$ applied to spruce wood in different directions.

#### 2.2.2. Grout

**Figure 2:**
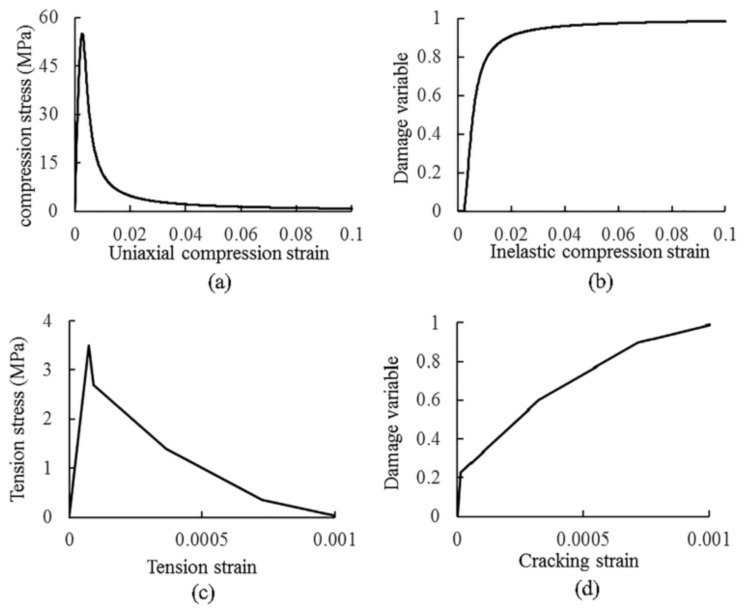
Material constitutive laws for cementitious grout: (**a**) stress-strain under compression; (**b**) compressive damage parameter versus inelastic strain; (**c**) stress-strain under tension; (**d**) tension damage parameter versus inelastic strain [14].

The mechanical properties of the cementitious grout reported in literature [14] were adopted directly in this study, since the same cementitious grout was used. Considering that both the regular concrete and the grout herein are cement-based materials, the concrete damage plasticity (CDP) model [21] was applied to model the grout in this study. The compressive strength is 55MPa, the modulus of elasticity is 37 GPa, Poisson’s ratio is 0.2, the flow potential eccentricity is 0.1, dilation angle is 38°, and a biaxial to uniaxial compressive strength ratio of 1.16 was used. The constitutive laws of cementitious grout are referenced in the literature [14], as shown in Figure 2.

#### 2.2.3. Steel Components

**Figure 3:**
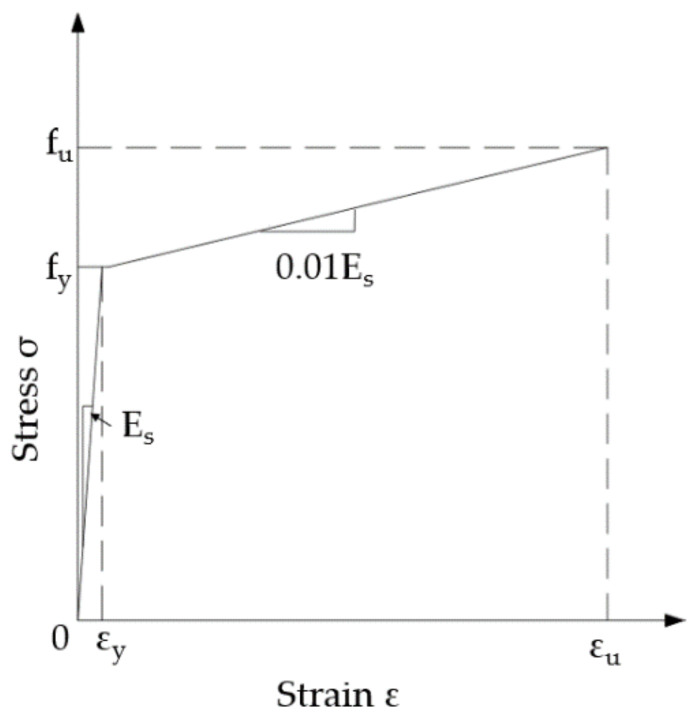
Idealized elastoplastic model for steel.

For all the steel components, including the steel beams and the studs, a linear strengthening elastoplastic model was used, as shown in Figure 3, where $f_{y}$ and $\varepsilon_{y}$ represent yield strength and yield strain, respectively, $f_{u}$ and $\varepsilon_{u}$ represent ultimate strength and its corresponding strain, respectively, and $E_{S}$ is modulus of elasticity. Von Mises yield criterion was adopted for modeling, and the nominal stress and strain were used. The steel beams were made of Q235 steel with a yield strength of 235 MPa and an ultimate strength of 375 MPa according to GB 50917-2013 [22]. The stud connectors were made of ML15 steel with a yield strength of 332.5 MPa and ultimate strength of 427 MPa, which meet the demands of GB/T 10433-2002 [23]. The modulus of elasticity and Poisson’s ratio are 210 GPa and 0.3, respectively.

### 2.3. Interactions and Boundary Conditions

The interactions in the FE models involve the following interfaces, namely grout–CLT, steel beam–CLT, and steel beam–grout. The tangential behavior at the above-mentioned interfaces was empirically defined by “Penalty” function with a friction coefficient of 0.1, 0.3, and 0.2, respectively, referring to [14]. The normal behavior at those interfaces was defined as “Hard” contact. The “Hard” contact in normal behavior and “Penalty” function in tangential behavior were adopted in the interactions between the surface of the stud shank and the grout pocket, and the friction coefficient of 0.4 for modeling the tangential behavior was used as recommended in [24,25]. Normal behavior of “Hard” contact and tangential behavior of “Frictionless” were adopted between the stud head (including top and bottom surface) and the grout pocket [26]. In addition, “Tie” contact was adopted between the studs and the steel beam [27].

### 2.4. Element Type and Meshing

**Figure 4:**
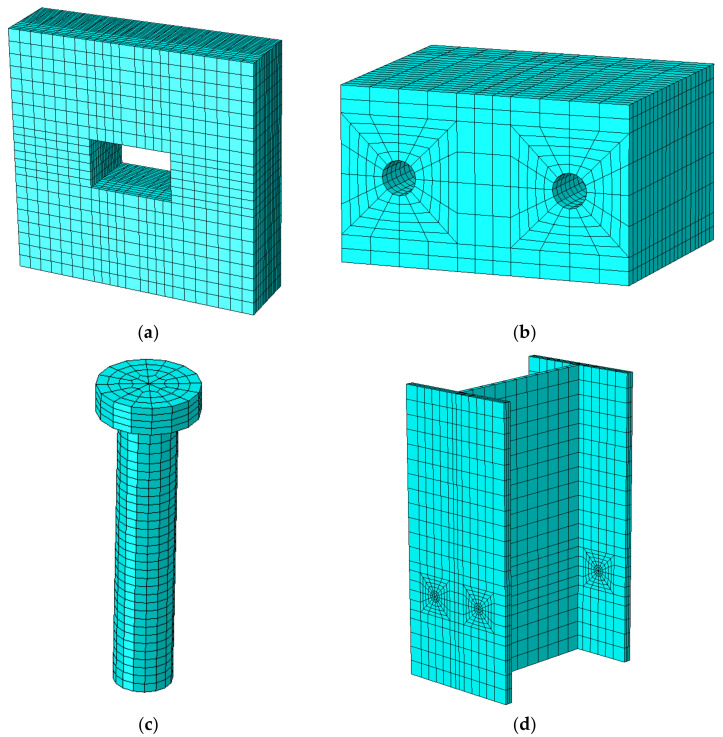
Meshing of components: (**a**) cross-laminated timber (CLT) panel; (**b**) grout; (**c**) stud connector; (**d**) steel beam.

All the components in FE models were meshed using 3D solid element with 8-node (C3D8R) linear hexahedron reduced integral scheme. To balance the accuracy of calculation and computational time, meshing was only locally refined at some areas, especially at the contact surfaces between grout pocket and stud and stud and steel beam. In addition, the mesh size of studs in the FE models were set as around 5 mm. Figure 4 shows the meshing of the components in the FE models.

### 2.5. Verification

**Figure 5:**
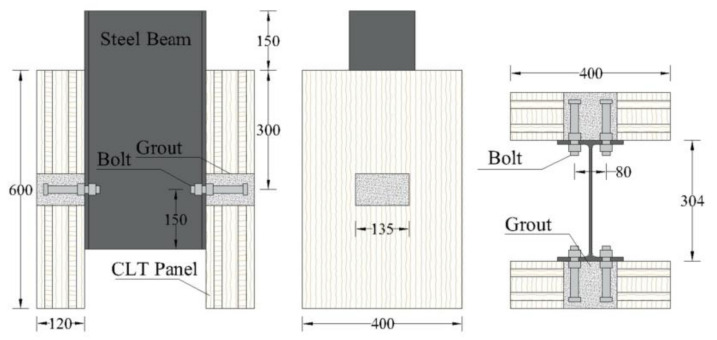
Bolt connectors embedded in grout pocket (BCGP) connections (units: mm) [13].

**Figure 6:**
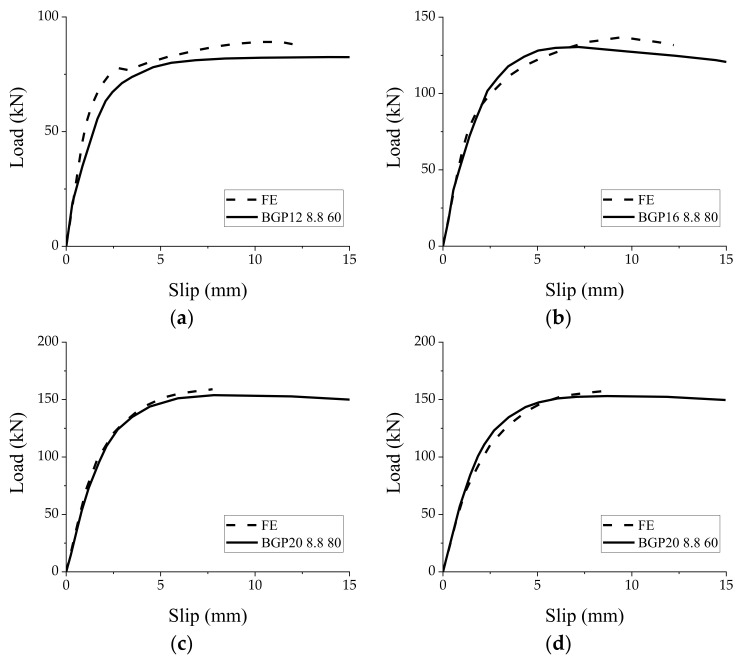
Comparison between simulated results and test curves from literature [13]. (**a**) BGP12 8.8 60; (**b**) BGP16 8.8 80; (**c**) BGP20 8.8 80; (**d**) BGP20 8.8 60.

**Figure 7:**
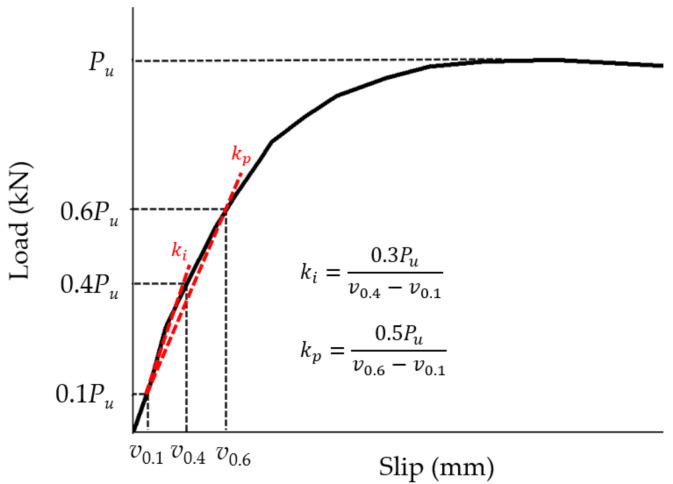
Definitions of initial and prepeak stiffness.

Before doing simulations on the GSC connections, several BCGP connections [13], as shown in Figure 5, were taken as examples to verify the reliability of FE models. Figure 6 shows the comparison between the simulated and experimental load–slip responses of the BCGP connections. Figure 6 shows that the FE load–slip curves are generally in good agreement with the experimental load–slip responses. The peak load capacity, initial stiffness, and pre-peak stiffness defined in Figure 7 are compared in Table 3. $k_{i}$ is defined as the secant modulus between 10% and 40% of the ultimate load carrying capacity ( $F_{u}$ ) for serviceability limit state, and $k_{p}$ is evaluated by that between 10% and 60% of $F_{u}$ for ultimate limit state [13,28]. As shown in Table 3, the difference of peak load capacity between the test results and the FE values is less than 7.5%. However, there are some errors between the simulated stiffness and experimental results, which might be due to the existing damage in wood panels due to cutting and drilling [14].

## 3. Parametric Study

### 3.1. Stud Diameter

**Figure 8:**
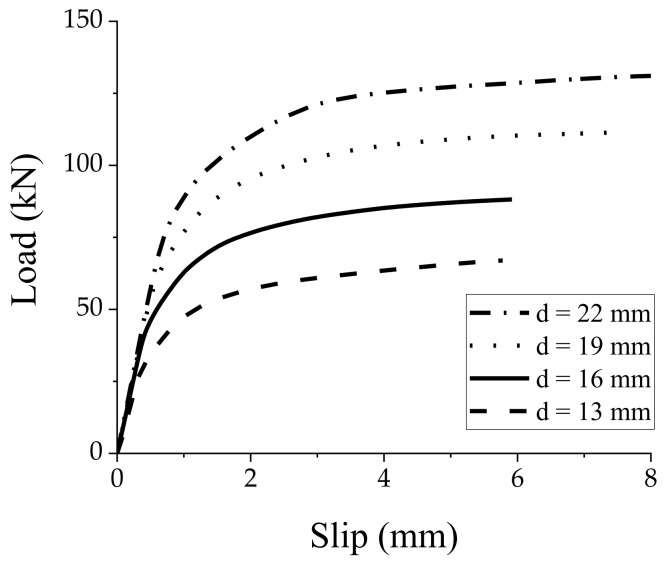
Load-slip responses of GSC connections with different stud diameters.

**Figure 9:**
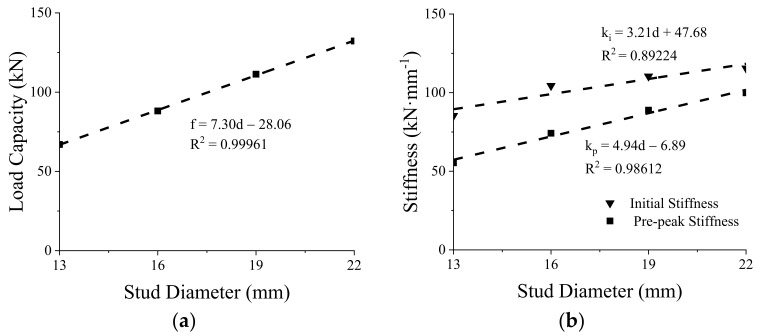
Peak load capacity (**a**) and stiffness (**b**) versus stud diameters.

The stud diameter is one of the most influential parameters on the mechanical properties of the shear connections [29,30]. Therefore, the effect of stud diameter on the shear performance of the GSC connections was evaluated by FE modeling. The studs with diameters of 13, 16, 19, and 22 mm were considered in the simulations. Figure 8 shows that the FE load–slip curves are characterized by an initial linear response followed by a nonlinearly yielded branch, which indicates that the larger the diameter, the higher the shear capacity of the GSC connections. Specifically, the shear capacity of the studs with a diameter of 16, 19, and 22 mm is 31.61%, 66.23%, and 97.45% higher than that of the 13-mm diameter stud, respectively. Figure 9 shows the relationships between three variables (initial stiffness, prepeak stiffness, and peak load capacity) and the stud diameters. Based on linear regression analysis, the relationship between the peak load capacity f  (kN) and stud diameter is obtained as

(1) f=7.30d−28.06

The relationship between the initial stiffness $k_{i}$ (kN∙mm^−1^), prepeak stiffness $k_{p}$ (kN∙mm^−1^), and stud diameter are expressed as follows, based on linear regression analysis:

(2) $$\left\{\begin{array}{l} k_{i}=3.21d+47.68\\ k_{p}=4.94d-6.89 \end{array}\right.$$

In Equations (1) and (2), d is the diameter of stud (Unit: mm).

**Figure 10:**
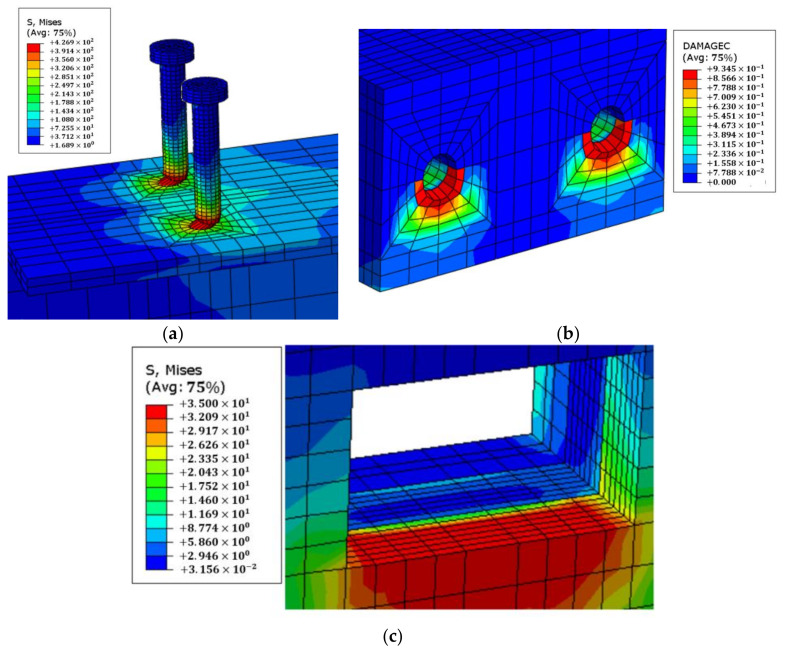
Failure mode of GSC connection: (**a**) stress nephogram of stud; (**b**) compressive damage of grout; (**c**) stress nephogram of CLT.

Typical failure modes of the GSC connections were analyzed by taking the connections with 16 mm diameter stud as an example, as shown in Figure 10. The GSC connections mainly failed by the yielding of studs and shear failure occurred (Figure 10a). The stress concentration on the grout was founded near the stud root, indicating local crushing of grout (Figure 10b). Moreover, obvious stress concentration of CLT panel at the compressive side of the grout pocket was also observed clearly, as shown in Figure 10c.

### 3.2. Stud Strength

**Figure 11:**
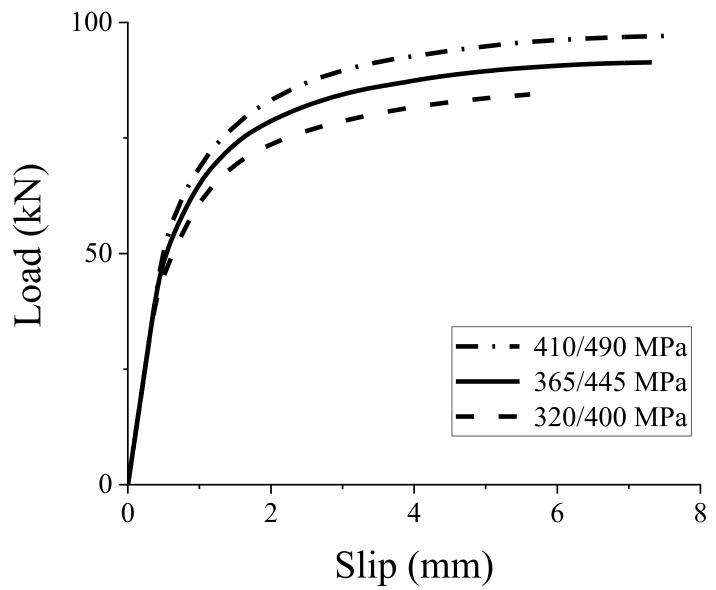
Effect of stud strength on load–slip response of GSC connections.

In this study, the studs with a yield strength of 320, 365, and 410 MPa are considered in the FE simulations, and their corresponding tensile strengths are 400, 445, and 490 MPa, respectively. Load–slip curves of the GSC connections with different strength are shown in Figure 11, and the peak load capacities are compared in Table 4. The peak load capacity and pre-peak stiffness of GSC connections present an upward trend with the increase in stud strength, while it has a slight influence on the initial stiffness.

### 3.3. Angle of Outer Layer of CLT Panel to Loading Direction

**Figure 12:**
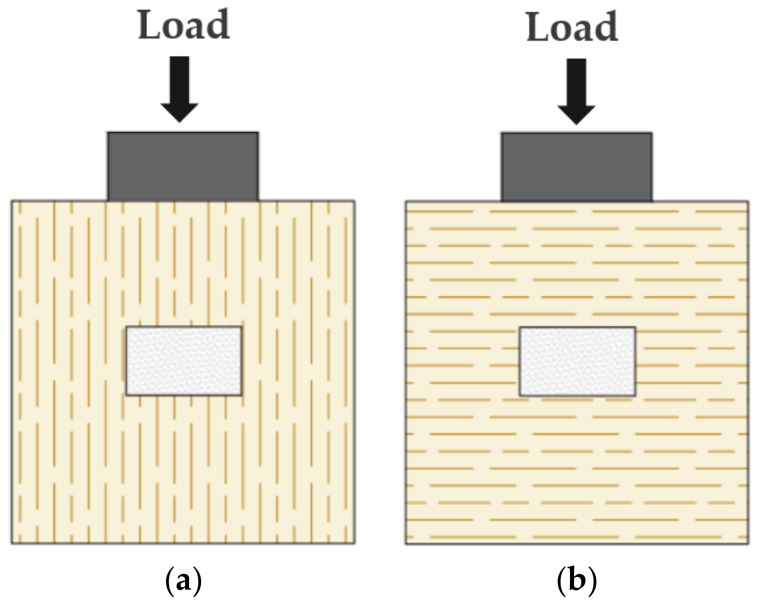
Angle of CLT outer layer parallel (**a**) and perpendicular (**b**) to loading direction.

**Figure 13:**
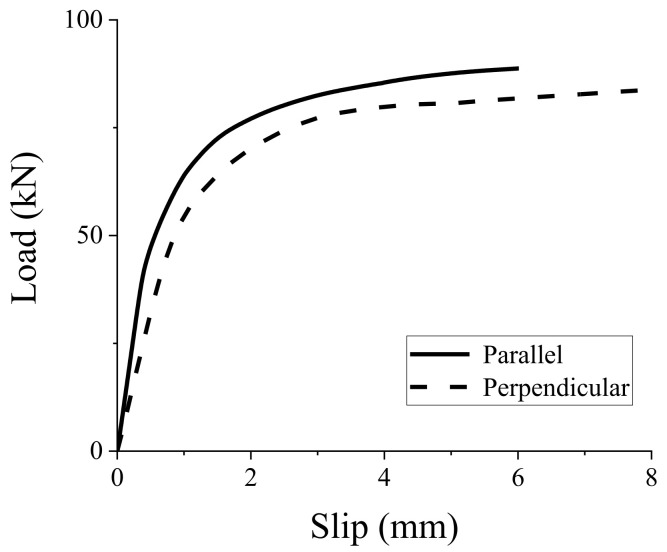
Effect of grain directions on load–slip response of GSC connections.

Previous experimental studies [13] indicated that the angle of the outer layer of CLT panel to loading direction has a significant effect on the stiffness of shear connectors. Accordingly, the GSC connection specimens with the CLT outer layers parallel and perpendicular to the loading direction were simulated (Figure 12). There is an obvious difference between the two curves in Figure 13, indicating that the angle of the outer layer of the CLT panel to loading direction has an influence on the load–slip behavior of the connections. Table 5 shows that the initial stiffness and pre-peak stiffness of the GSC connections with the CLT outer layer perpendicular to the loading direction decreased by 38.4% and 23.3% compared to that parallel to the load direction, respectively. This is mainly due to the relatively lower modulus of elasticity of laminates perpendicular to grain compared to that parallel to the grain. Table 5 also shows that the angle of the outer layer of the CLT panel to loading direction has a slight influence (around 5%) on the peak load capacity of the simulated connections.

### 3.4. Tapered Configurations of Grouting Groove

**Figure 14:**
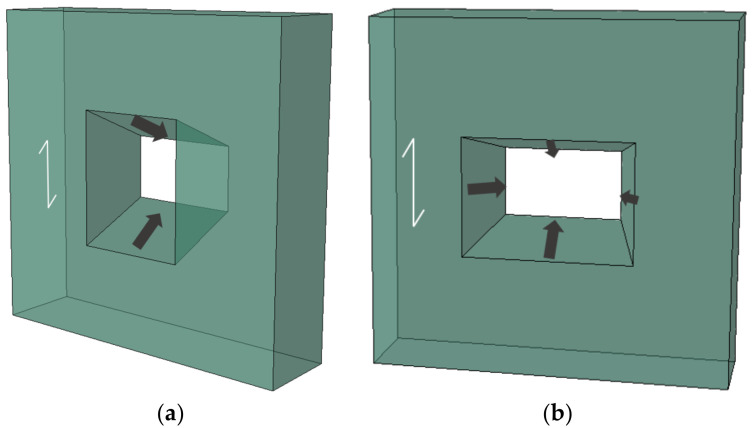
Tapered groove configurations: (**a**) unidirectional; (**b**) bidirectional.

**Figure 15:**
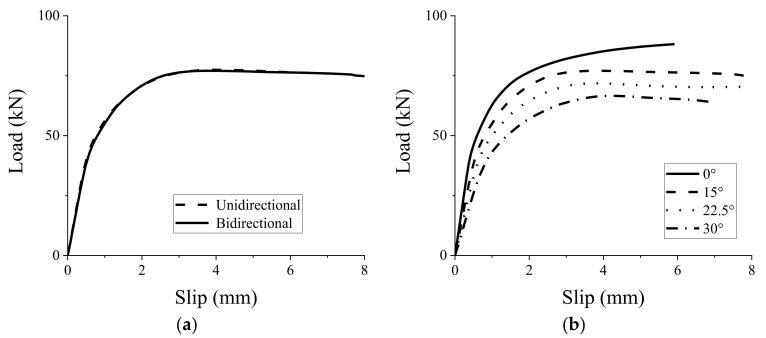
Effect of different groove parameters on load–slip response of GSC connections: configurations (**a**) and angles (**b**).

**Figure 16:**
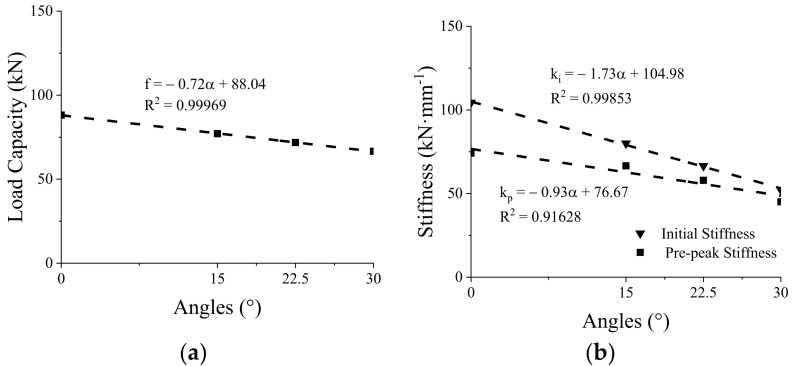
Peak load capacity (**a**) and initial stiffness (**b**) versus angles of grouting groove.

In composite beams, the shear connectors need to resist the uplift effect between the upper slab and the bottom beam, as well as transmit the shear flows between the two components. Tapered configurations for grouting grooves should be an effective way to improve the uplift resistance of the GSC connections. Thus, several types of tapered groove configurations (15°, 22.5°, and 30°) were designed herein to evaluate the shear performance and uplift resistance of the GSC connections. Noting that for the angle of 15°, unidirectional and bidirectional tapered groove configurations were considered, as shown in Figure 14. Figure 15 shows the effect of the angle of the tapered configurations on the load–slip response of the GSC connections. Figure 15a shows that the GSC connections with unidirectional and bidirectional tapered configurations almost show the same load–slip behaviors, indicating that the unidirectional and bidirectional tapered groove has a negligible effect on shear behavior of the GSC connections. Thus, in real applications, unidirectional tapered grooves are recommended for the GSC connections due to their convenience of construction. Figure 15b and Table 6 show the FE simulated results of the GSC connections with unidirectional tapered grooves with different angles. Obviously, the angle of the tapered groove shows significant influence on both the peak load capacity and the stiffness. Specifically, as the angle of grouting groove increased from 0° to 30°, both the peak load capacity and stiffness decrease generally in an approximately linear proportional relationship, as shown in Figure 16. The linear relationships between the peak load capacity f (kN) and the angle of tapered groove as well as between the initial stiffness $k_{i}$ (kN∙mm^−1^) and pre-peak stiffness $k_{p}$ (kN∙mm^−1^) can be expressed as follows.

(3) f=−0.72α+88.03

(4) $$\left\{\begin{array}{l} k_{i}=-1.73\alpha +104.98\\ k_{p}=-0.93\alpha +76.67 \end{array}\right.$$

In Equations (3) and (4), α is the angle of tapered groove.

**Figure 17:**
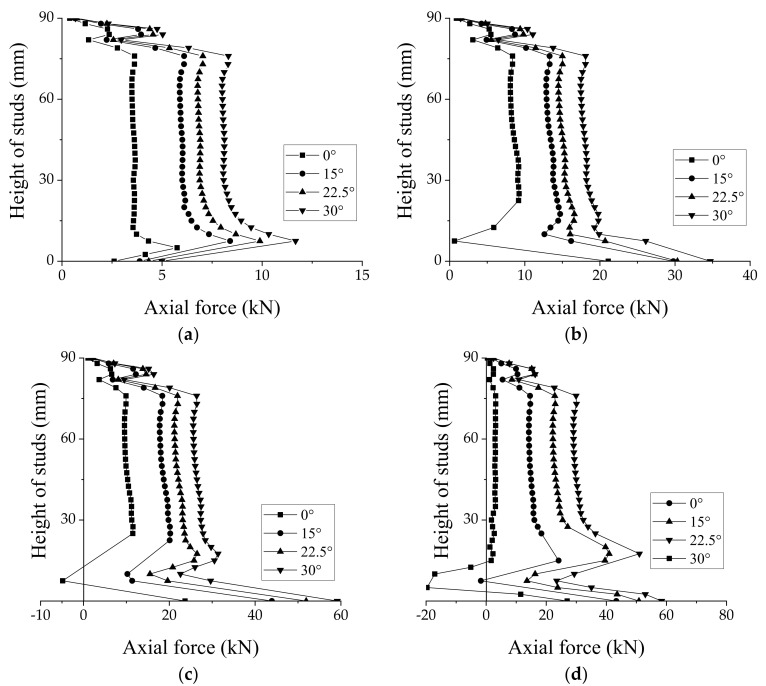
Axial force along length of studs in GSC connections with different angles of groove at (**a**) 0.2 $F_{max}$ , (**b**) 0.5 $F_{max}$ , (**c**) $F_{y}$ , and (**d**) $F_{max}$ .

Figure 17 depicts the FE simulated axial force of the studs in the GSC connections with different angles of grouting groove at different load levels (0.2 $F_{max}$ , 0.5 $F_{max}$ , $F_{y}$ , $F_{max}$ ). $F_{max}$ and $F_{y}$ are the maximum load and yield load of the connections, respectively. Generally, at the same load level, the larger the angle of the tapered groove, the higher the axial force of the studs; therefore, using the tapered groove can improve the uplift resistance of the GSC connections effectively. Figure 17 shows that stress concentration phenomenon is clearly observed near the root of the studs, especially at the load level of $F_{y}$ and $F_{max}$ .

### 3.5. Thickness of CLT Panel

**Figure 18:**
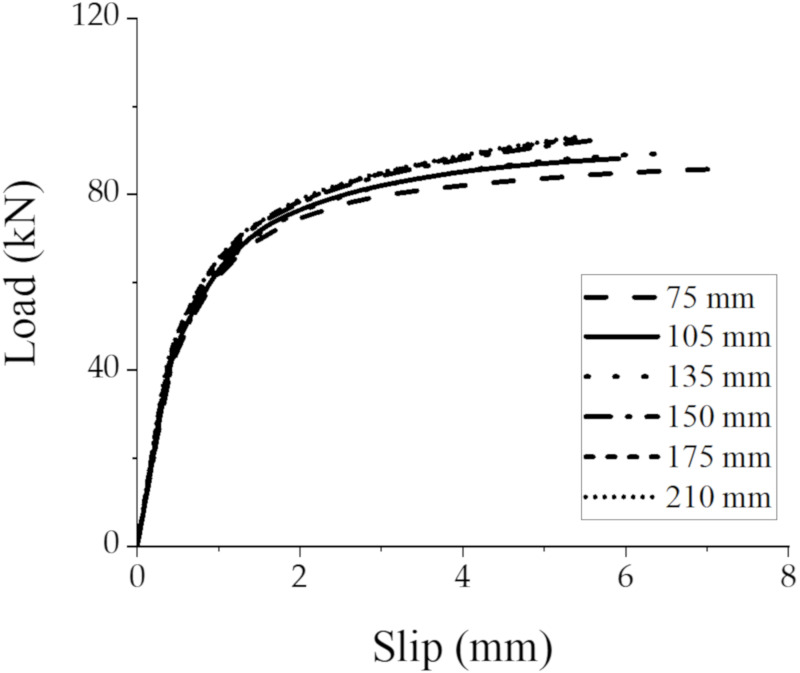
Load–slip responses of GSC connections with different thicknesses of CLT panel.

Considering the available regular thicknesses of CLT panels in the market, this paper investigated GSC connections with 75 (3 × 25 mm)-, 105 (3 × 35 mm)-, 135 (9 × 15 mm)-, 150 (5 × 30 mm)-, 175 (5 × 35 mm)-, and 210 mm (7 × 30 mm)-thick CLT panels, which in parentheses represent the number and the thickness of layers of CLT panels. The load–slip responses are shown as Figure 18. The peak-load capacities of the GSC connections with different thicknesses of CLT panel increased from 85.78 kN to 93.15 kN, with the thickness of CLT panel increased from 75 to 210 mm indicating that the bearing capacity of GSC connections has a minor improvement as the CLT thickness increased from 75mm to 150 mm, while the capacity almost has no increases when the thickness reached 150 mm. In general, the thickness of CLT panel has a slight influence on the shear capacity of the GSC connections.

## 4. Calculations of Shear Capacity

To some degree, the shear force transmission between the upper slab and the bottom beam in STC system is quite similar to that of the stud connectors in SCC beams. Therefore, the available calculation formulas for the shear capacity of stud in SCC beams were adopted to predict the shear capacity of GSC connections herein.

In the *Standard for Design of Steel Structures* (GB 50017-2017) [22], the shear capacity of an individual stud connector is calculated as:

(5) $$N_{v}^{c}=0.43A_{s}\sqrt{E_{c}f_{c}}\leq 0.7A_{s}f_{u}$$

where, $N_{v}^{c}$ is the shear capacity (N) for individual stud; $E_{c}$ and $f_{c}$ are the modulus of elasticity and compressive strength of concrete (MPa); $A_{s}$ is the cross-sectional area of the shank of stud (mm^2^); and $f_{u}$ is the tensile strength of stud (MPa).

According to the *Code for Design of Steel and Concrete Composite Bridges* (GB50917-2013) [31], the shear capacity of a single stud connector should take the smaller value in Equation (6):

(6) $$\left\{\begin{array}{l} N_{v}^{c}=1.19A_{std}f_{std}{\left(\frac{E_{c}}{E_{s}}\right)}^{0.2}{\left(\frac{f_{cu}}{f_{std}}\right)}^{0.1}\\ N_{v}^{c}=0.43\eta A_{std}\sqrt{f_{cd}E_{c}} \end{array}\right.$$

where, $N_{v}^{c}$ is the shear capacity (N); $A_{std}$ is the cross-sectional area of the shank of stud (mm^2^); $E_{c}$ and $E_{s}$ are the modulus of elasticity of concrete and steel (MPa); $f_{cu}$ is the cubic compressive strength of concrete (MPa); $f_{cd}$ is the design value of axial compressive strength of concrete (MPa); $f_{std}$ is the tensile strength of stud (MPa); and η is the reduced coefficient of group effect.

The design shear resistance of a stud in Eurocode 4 [32] is reported to be taken as the minimum value in the following two formulas:

(7) $$\left\{\begin{array}{l} P_{Rd}=0.29\alpha d^{2}\sqrt{f_{ck}E_{cm}}/\gamma_{V}\\ P_{Rd}=0.8A_{d}f_{u}/\gamma_{V} \end{array}\right.$$

where, $P_{Rd}$ is the shear capacity (N); α=1 for $h_{sc}/d>4$ ; d is the diameter of the shank of the stud (mm); $h_{sc}$ is the overall nominal height of the stud; $E_{cm}$ is the modulus of elasticity of concrete (MPa); $f_{ck}$ is the characteristic cylinder compressive strength of the concrete (MPa); $A_{d}$ is the cross-sectional area of the shank of stud (mm^2^); $f_{u}$ is the ultimate tensile strength of stud (MPa); $\gamma_{V}$ is the partial factor; and the recommended value is 1.25.

The *Specification for Structural Steel Buildings* (ANSI/AISC 360-16) [33] presents that the shear strength of one stud should be determined as follows:

(8) $$Q_{n}=0.5A_{sa}\sqrt{f_{c}^{’}E_{c}}\leq R_{g}R_{p}A_{sa}F_{u}$$

where $Q_{n}$ is the shear capacity (N); $A_{sa}$ is the cross-sectional area of the stud shank (mm^2^); $f_{c}^{’}$ is the specified compressive strength of concrete (MPa); $E_{c}$ is the modulus of elasticity of concrete (MPa); $F_{u}$ is the specified minimum tensile strength of stud (MPa); $R_{g}\;$ = 0.85; and $R_{p}\;$ = 0.75.

Ding et al. [26] established a calculation formula for the shear capacity of an individual stud based on the push-out test results of stud connectors in worldwide, expressed as Equation (9):

(9) $$P_{u}=\left(0.2d^{1.7}-10\right)f_{cu}^{0.8-0.15\ln\left(d-10\right)}\left(0.002f_{y}+0.24\right)$$

where $P_{u}$ is the shear capacity (N); d is the diameter of the stud shank (mm); $f_{cu}$ is cubic compressive strength of concrete (MPa); and $f_{y}$ is the yield strength of stud (MPa).

Zhou et al. [34] proposed a formula for calculating the shear capacity of one stud by regression analysis of the push-out test data of 233 stud connectors in worldwide, as shown in Equation (10):

(10) $$V_{u}=\left\{\begin{array}{l} 0.5A_{s}\sqrt{E_{c}f_{ck}},A_{s}\sqrt{E_{c}f_{ck}}\leq 344,000N\\ 0.21A_{s}\sqrt{E_{c}f_{ck}}+165A_{s},A_{s}\sqrt{E_{c}f_{ck}}>344,000N \end{array}\right.$$

where $V_{u}$ is the shear capacity (N); $A_{s}$ is the cross-sectional area of the shank of the stud (mm^2^); $E_{c}$ is the modulus of elasticity of concrete (MPa); and $f_{ck}$ is prism compressive strength of concrete (MPa).

Zhang [30] made a regression analysis of the push-out test data of 80 stud connectors and proposed a calculation model for the shear capacity of a single stud connectors as follows:

(11) $$V_{u}=17.31A_{s}f_{u}{\left(\frac{h_{s}}{d_{s}}\right)}^{0.27}{\left(\frac{E_{c}}{E_{s}}\right)}^{1.75}{\left(\frac{f_{cu}}{f_{u}}\right)}^{0.14}$$

where $V_{u}$ is the shear capacity (N); $A_{s}$ is the cross-sectional area of the shank of the stud (mm^2^); $E_{c}$ and $E_{s}$ are the modulus of elasticity of concrete and steel (MPa); $f_{cu}$ is the cubic compressive strength of concrete (MPa); and $f_{u}$ is the ultimate tensile strength of stud (MPa).

Wang [35], suggested the following formula for calculating the shear capacity of one stud based on linear regression analysis of the push-out test values of stud connectors:

(12) $$P_{u}=3A_{s}f{\left(\frac{E_{c}}{E_{s}}\right)}^{0.4}{\left(\frac{f_{cu}}{f}\right)}^{0.2}$$

where $P_{u}$ is the shear capacity (N); $A_{s}$ is the cross-sectional area of the shank of the stud (mm^2^); $E_{c}$ and $E_{s}$ are the modulus of elasticity of concrete and steel (MPa); $f_{cu}$ is the cubic compressive strength of concrete (MPa); and f is the yield strength of stud (MPa).

The concrete strength in all the above calculation modes is directly substituted by the corresponding strength of grout in the pockets.

**Figure 19:**
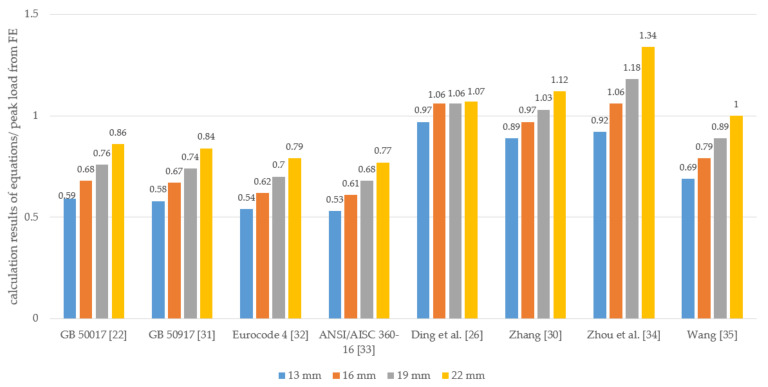
Comparison between simulated peak load and predictions from existing calculation modes.

Figure 19 shows the comparison between the predicted shear capacity of the GSC connections from the abovementioned calculation formulas and the FE simulated results. Generally, the calculation modes in the design codes (i.e., Eurocode 4, ANSI/AISC 360-16, GB 50017, and GB 50917) conservatively estimate the shear capacity of the GSC connections compared to that of the FE simulated results. Further, the shear capacity predicted by the formulas (Ding et al., Zhang, Zhou et al., and Wang) is generally in good agreement with the simulation results; in particular, the formulas from Ding et al. and Zhang [26,30] show a difference of less than 12% compared to the simulation values. Thus, the Ding et al. and Zhang calculation modes are suggested for predicting the shear capacity of the GSC connections for STC systems.

## 5. Group Effect

Since the GSC shear connections in STC beams are probably composed of a group of studs, the group effect of the stud group is deserved to be discussed. Theoretically, the stud group has a discounted shear capacity compared to the sum of the shear capacity of individual stud, which can be illustrated by a reduce factor. To obtain this reduce factor, The influence of group effect on the shear properties of the GSC connections was evaluated by FE modeling.

**Figure 20:**
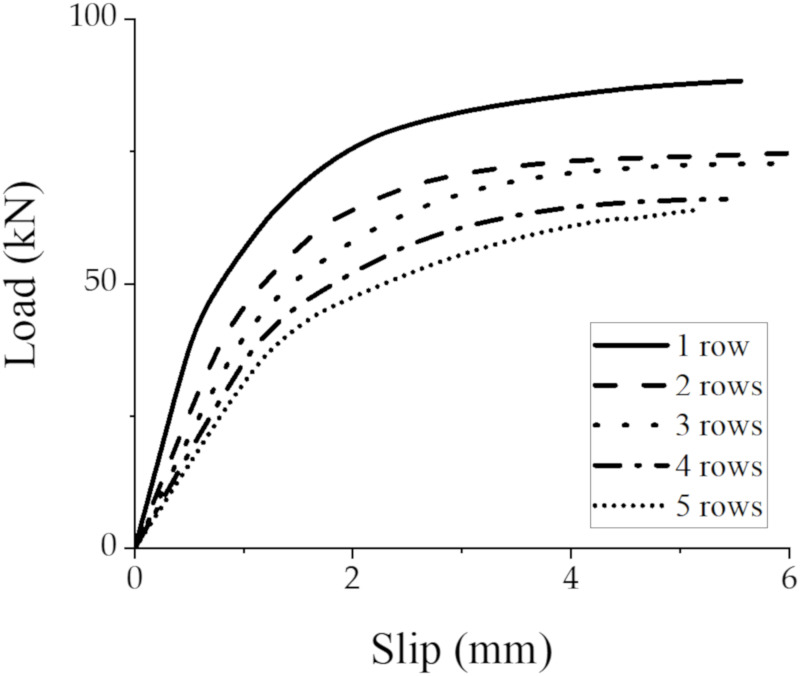
Load–slip response of individual stud of GSC connections with different rows of studs.

The GSC connections with 2–5 rows of studs with a row spacing of 48 mm (3d) were designed and compared to the GSC connection with a single row of studs; the stud diameter is 16mm. Figure 20 shows the comparison of load–slip responses of individual stud between the connections with 1–5 rows of studs. Table 7 shows the averaged shear capacity, initial stiffness, and pre-peak stiffness of the GSC connections with different rows of studs. Generally, the shear capacity, initial stiffness, and pre-peak stiffness decrease gradually as the stud row increased from one to five, confirming the influence of group effect in the average shear strength and stiffness of individual stud.

**Figure 21:**
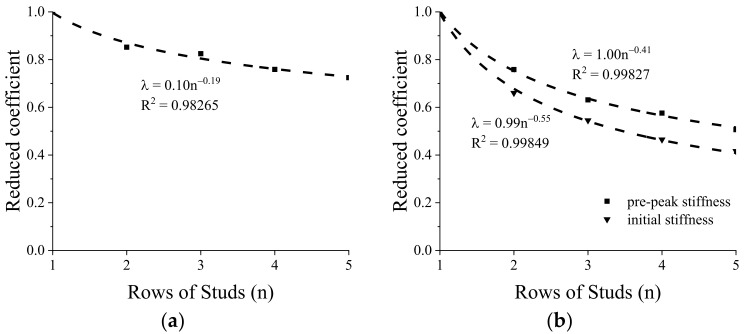
Reduced coefficient of stud rows for peak-load capacity (**a**) and stiffness (**b**) of GSC connections with different rows of studs.

Such a performance degradation can be described by the concept of effective row number ( $n_{eff}$ ), which is obtained by reducing the rows of studs ( n ). In this paper, the reduced coefficient of stud rows ( λ ) was obtained by regression analysis of the simulation results (Figure 21), and $n_{eff}=\lambda \times n$ . When evaluating the peak load capacity, the group effect can be expressed as:

(13) $$n_{eff}=0.10n^{0.81}$$

For initial stiffness and pre-peak stiffness, the group effect can be described as:

(14) $$\left\{\begin{array}{l} n_{eff,i}=0.99n^{0.45}\\ n_{eff,p}=n^{0.59} \end{array}\right.$$

## 6. Conclusions

In this study, the GSC connections are proposed for the connections between the timber slab and the steel beam in steel–timber composite (STC) system. The shear performance of the GSC shear connections was studied by FE modeling, and parametric analyses were also conducted. Moreover, the FE simulated shear capacity was compared with the results predicted by the available calculation formulas in design codes and literature. Based on the numerical works, the following conclusions can be drawn:

- The load–slip response of the GSC connections obtained by FE modeling is characterized by an initial linear increase response followed by a nonlinearly yielded branch. The stud diameter has a significant effect on the shear performance of the GSC connections. With the increase in stud diameter from 13 to 22 mm, the peak load capacity and stiffness of the GSC connections increased almost linearly.
- The stud strength has a certain influence on the shear performance of the GSC connections. The peak load capacity and pre-peak stiffness of the GSC connections increase with the growth of stud yield strength from 320 to 410 MPa and the tensile strengths from 400 to 490 MPa correspondingly, while it has a minor effect on the initial stiffness.
- The unidirectional and bidirectional tapered grooves have a negligible effect on shear behavior of the GSC connections, and the unidirectional tapered grooves are recommended due to their convenience of construction. The unidirectional tapered groove with different angles has a considerable influence on the shear capacity and stiffness of the GSC connections. With the increase in groove angles from 0°to 30°, the peak load capacity and stiffness decrease. Using unidirectional tapered grooves with an appropriate angle (15°) is suggested for improving the uplift resistance of the GSC connections.
- The shear capacity predicted by the formulas (Ding et al., Zhang, Zhou et al., and Wang) is generally in good agreement with the simulation results. However, the calculation modes in the design codes underestimate the shear capacity of the GSC connections compared to that of the FE simulated results.
- The peak load capacity and stiffness of individual studs in GSC connections are obviously reduced due to the group effect. The average value of peak load capacity and stiffness of individual studs gradually decrease with the increase in the rows of studs from 1 to 5.

## Figures and Tables

**Table 1:** Elastic moduli and Poisson’s ratios applied to spruce wood lamellae (MPa) [14].

Modulus of Elasticity			Poisson’s Ratio			Shear Modulus		
$E_{11}$	$E_{22}$	$E_{33}$	$V_{12}$	$V_{13}$	$V_{23}$	$G_{12}$	$G_{13}$	$G_{23}$
11,000	370	370	0.48	0.48	0.22	690	690	50

**Table 2:** Compressive strength $f_{c}$ , tensile strength $f_{t}$ , shear strength $f_{s}$ , and fracture energies $G_{f}$ of spruce wood [14].

Parallel-to-Grain (MPa)		Perpendicular-to-Grain (MPa)		Shear Strength (MPa)		Fracture Energy (N·mm^−1^)			
$f_{c11}$	$f_{t11}$	$f_{c22}$	$f_{t22}$	$f_{s}$	$f_{s\;roll}$	$G_{f,0}$	$G_{f,90}$	$G_{f,v}$	$G_{f,roll}$
35	24	4.3	0.7	6.9	0.5	6.0	0.5	1.2	0.6

**Table 3:** Comparison of stiffness and strength from finite element (FE) models with test data from literature [13].

Specimen	BGP12 8.8 60			BGP16 8.8 80			BGP20 8.8 80			BGP20 8.8 60		
	Exp.	FE	Error (%)	Exp.	FE	Error (%)	Exp.	FE	Error (%)	Exp.	FE	Error (%)
Initial Stiffness (kN∙mm^−1^)	39.53	53.99	36.58	55.12	61.45	11.48	77.19	67.78	12.19	64.57	60.82	5.81
Pre-peak Stiffness (kN∙mm^−1^)	29.76	48.86	64.18	45.77	48.22	5.35	63.08	58.17	7.78	53.68	47.02	12.41
Peak load capacity (kN)	82.9	89.11	7.49	129.9	136.70	5.23	159.7	159.09	0.38	153.3	156.89	2.34

**Table 4:** Effect of stud strength on peak load capacity of GSC connections.

Yield Strength/Tensile Strength (MPa)	320/400	365/445	410/490
Initial Stiffness (kN∙mm^−1^)	104.26	105.04	105.48
Pre-peak Stiffness (kN∙mm^−1^)	73.54	79.52	85.68
Peak load capacity (kN)	84.43	91.36	97.07

**Table 5:** Effect of grain directions on peak load capacity and stiffness of the GSC connections.

Grain Directions	Parallel	Perpendicular	Difference * (%)
Initial Stiffness (kN∙mm^−1^)	104.39	64.28	38.4%
Prepeak Stiffness (kN∙mm^−1^)	74.18	56.86	23.3%
Peak load capacity (kN)	88.16	83.76	5.0%

* Difference = (Parallel–Perpendicular)/Parallel.

**Table 6:** Effect of groove angles on stiffness and peak load capacity of GSC connections.

Angles of Grouting Groove	0°	15°	22.5°	30°
Initial Stiffness (kN∙mm^−1^)	104.39	79.98	66.52	52.24
Prepeak Stiffness (kN∙mm^−1^)	74.18	66.56	57.91	45.10
Peak load capacity (kN)	88.16	77.05	71.82	66.61

**Table 7:** Group effect on peak load capacity and stiffness of GSC connections.

Rows	1	2	3	4	5
Initial Stiffness (kN∙mm^−1^)	76.12	50.24	41.49	35.37	31.69
Pre-peak Stiffness (kN∙mm^−1^)	57.62	43.70	36.37	33.18	29.18
Peak load capacity (kN)	88.30	75.24	72.84	67.0	63.95

## Data Availability

Not applicable.
