# Correction: Zhang, H.; Ling, Z. Finite Element Modeling on Shear Performance of Grouted Stud Connectors for Steel–Timber Composite Beams. *Materials* 2022, *15*, 1196

**DOI:** 10.3390/ma15072362

**Published:** 2022-03-23

**Authors:** Henan Zhang, Zhibin Ling

**Affiliations:** School of Civil Engineering, Suzhou University of Science and Technology, Suzhou 215011, China; 1913031088@post.usts.edu.cn

The authors wish to make the following corrections to the published paper [1]. They should be as follows:

**Missing Funding**

In the original publication, the funder **Postgraduate Research & Practice Innovation Program of Jiangsu Province**, **SJCX20_1116** to **Henan Zhang and Zhibin Ling** was not included. The authors apologize for any inconvenience caused and state that the scientific conclusions are unaffected. This correction was approved by the Academic Editor. The original publication has also been updated.
